# The New York Code

**Published:** 1882-07

**Authors:** F. B. Cosford

**Affiliations:** Secretary


### The New York Code.

At the annual meeting of the Onondaga Medical Society, held June 13th, the following resolutions were adopted by the following vote—twenty in favor, six opposed, and four declining to vote:

WHEREAS, The code of ethics of the American Medical Association, promulgated in 1847, was soon afterwards adopted by the Medical Society of the State of New York, and afterward by the Onondaga Medical Society; by a provision of this code consultation with practitioners who profess to be governed by an exclusive dogma is forbidden; it is the opinion of the Onondaga Medical Society that the State Society in assuming to repeal this provision and to sanction consultations with anybody and everybody exceeded its powers and justly forfeited all right to representation in the American Medical Association.

WHEREAS, It is also the opinion of the Onondaga Medical Society that the amendment to the code adopted by the State Society is impolitic, unwise, hurtful to the interests of the profession and of the public; and that it merits, as it seems to receive, general reprobation. It is as silly to charge the regular medical profession with illiberality and want of progress because it will not consult with the advertising quack or the doctor who professes to give doses so small as to be beyond the ken of human imagination, as to make the same charge against the orthodox clergyman because he declines to invite the atheist to consult as to the best method of promoting evangelical religion. The regular profession is liberal, tolerating the wildest differences of opinion; it is progressive, ready to test and welcome every new discovery in science and in the healing art; it points with just pride to all the glorious achievements of the past fifty years, in sanitary science and pathology, in surgery and in measures for preventing and alleviating pain, and shortening safely the course of disease; and it claims every one as the outcome of its professional and investigating policy; therefore

*Resolved*, That the permanent members of the State Society from this county be requested, and the delegates be instructed, to use all proper means to secure the prompt repeal of the New Code, against which this Society protested at its winter meeting.

### RESOLUTIONS ADOPTED AT THE LAST MEETING OF THE NIAGARA CO. MEDICAL SOCIETY.

Dr. Gould presented the following resolutions expressing the feeling of Niagara County Medical Society in the course taken by the State Medical Society at its last meeting:

WHEREAS, A judicious code of medical ethics is essential to the best interests of the medical profession and tends to promote the welfare and harmony of its members, as also the public good, it would seem but the dictate of sound judgment and good taste that the code of ethics so long adopted by the American Medical Association, and by our State Medical Society, should not be changed, except on due notice of such intention, and ample time given for mature deliberation and exchange of the views and opinions held by all interested and affected thereby, to the end that any such change when made should reflect the sentiment of the great majority of the members of the profession, and tend to conserve the best interest of legitimate medicine and the kindred arts and science; therefore

*Resolved,* That the action of the State Medical Society at its last meeting (annual) in 1882 in reference to a change in the code of ethics is not in harmony with the sentiments of this society.

*Resolved,* That the delegates of this society to the State Society at its next annual meeting are hereby instructed to protest against said action of the State Society, and in all suitable ways seek to rescind the same.

F. B. COSFORD, *Secretary.*

---

